# Supplementary material for: The clinical efficacy and safety of equipment-assisted intravesical instillation of mitomycin C after transurethral resection of bladder tumour in patients with nonmuscular invasive bladder cancer: A meta-analysis
Source: PLoS One. 2022 Oct 21;17(10):e0276453. doi: 10.1371/journal.pone.0276453 (PMC9586381; doi:10.1371/journal.pone.0276453)
Supplement: S2 File — (PDF) [file pone.0276453.s002.pdf]

CNKI:

(((主题%= 非肌层浸润性膀胱癌+表浅膀胱癌+浅表型膀胱肿瘤+非肌层浸润性膀胱肿瘤+浅表性膀胱癌+表浅层膀胱癌+表浅性膀胱癌 or 题名%= 非肌层浸润性膀胱癌+表浅膀胱癌+浅表型膀胱肿瘤+非肌层浸润性膀胱肿瘤+浅表性膀胱癌+表浅层膀胱癌+表浅性膀胱癌) AND (旧版主题= 随机对照试验+随机+对照)) AND (主题%='丝裂霉素' or 题名%='丝裂霉素'))；检索范围：中文文献。

VIP:

((((((((题名或关键词=非肌层浸润性膀胱癌 OR 题名或关键词=superficial bladder cancer) OR 题名或关键词=表浅膀胱癌) OR 题名或关键词=浅表型膀胱肿瘤) OR 题名或关键词=非肌层浸润性膀胱肿瘤) OR 题名或关键词=浅表性膀胱癌) OR 题名或关键词=表浅层膀胱癌) OR 题名或关键词=表浅性膀胱癌) AND ((摘要=随机对照试验 OR 摘要=随机) OR 摘要=对照)) AND 题名或关键词=丝裂霉素)

WANFANG:

主题:(非肌层浸润膀胱癌+表浅膀胱癌+浅表型膀胱肿瘤+非肌层浸润性膀胱肿瘤+浅表性膀胱癌+表浅层膀胱癌+表浅性膀胱癌) and 主题:(丝裂霉素) and 摘要:(随机对照试验+随机+对照)

CBM：

序号　检索表达式　命中文献数　检索时间

1)　"膀胱肿瘤"[不加权:扩展]　78102　2022-04-12 18:25:18.0

2)　"膀胱癌"[常用字段:智能] OR "膀胱肿瘤"[常用字段:智能] OR "膀胱癌症"[常用字段:智能]　94014　2022-04-12 18:38:14.0

3)　("膀胱癌"[常用字段:智能] OR "膀胱肿瘤"[常用字段:智能] OR "膀胱癌症"[常用

字段:智能]) OR ("膀胱肿瘤"[不加权:扩展]) 94014 2022-04-12 18:38:43.0

4) "浅表"[常用字段:智能] OR "非肌层"[常用字段:智能] 21896 2022-04-12 18:41:14.0

5) ("浅表"[常用字段:智能] OR "非肌层"[常用字段:智能]) AND (("膀胱癌"[常用字段:智能] OR "膀胱肿瘤"[常用字段:智能] OR "膀胱癌症"[常用字段:智能]) OR ("膀胱肿瘤"[不加权:扩展])) 3224 2022-04-12 18:41:35.0

6) "丝裂霉素"[不加权:扩展] 15499 2022-04-12 18:45:42.0

7) "丝裂霉素 C"[常用字段:智能] 27584 2022-04-12 19:11:24.0

8) ("丝裂霉素 C"[常用字段:智能]) OR ("丝裂霉素"[不加权:扩展]) 27584 2022-04-12 19:11:36.0

9) "随机对照试验"[不加权:扩展] 499796 2022-04-12 19:12:37.0

10) "随机"[常用字段:智能] OR "对照"[常用字段:智能] 3445358 2022-04-12 19:12:52.0

11) ("随机"[常用字段:智能] OR "对照"[常用字段:智能]) OR ("随机对照试验"[不加权:扩展]) 3445358 2022-04-12 19:13:05.0

12) (("随机"[常用字段:智能] OR "对照"[常用字段:智能]) OR ("随机对照试验"[不加权:扩展])) AND (("丝裂霉素 C"[常用字段:智能]) OR ("丝裂霉素"[不加权:扩展])) AND (("浅表"[常用字段:智能] OR "非肌层"[常用字段:智能]) AND (("膀胱癌"[常用字段:智能] OR "膀胱肿瘤"[常用字段:智能] OR "膀胱癌症"[常用字段:智能]) OR ("膀胱肿瘤"[不加权:扩展]))) 144 2022-04-12 19:13:41.0

Pubmed:

("placebo"[Title/Abstract] OR "randomized"[Title/Abstract] OR "rct"[Title/Abstract] OR "Randomized Controlled Trial"[Publication Type]) AND ("Mitomycin-C"[Title/Abstract] OR "Mitomycin-C"[Title/Abstract] OR "Mitocin-C"[Title/Abstract] OR "Mitocin-C"[Title/Abstract] OR "NSC-26980"[Title/Abstract] OR "NSC-26980"[Title/Abstract] OR "NSC26980"[Title/Abstract] OR "Ametycine"[Title/Abstract] OR "Mutamycin"[Title/Abstract] OR "Mitomycin"[MeSH Terms]) AND (("non-muscle-invasive"[Title/Abstract] OR "superficial"[Title/Abstract] OR "nmibc"[Title/Abstract]) AND ("neoplasm urinary bladder"[Title/Abstract] OR "urinary bladder neoplasm"[Title/Abstract] OR "bladder tumors"[Title/Abstract] OR "bladder tumor"[Title/Abstract] OR "tumor bladder"[Title/Abstract] OR "tumors bladder"[Title/Abstract] OR "neoplasms bladder"[Title/Abstract] OR "bladder neoplasms"[Title/Abstract] OR "bladder neoplasm"[Title/Abstract] OR "neoplasm bladder"[Title/Abstract] OR "urinary bladder cancer"[Title/Abstract] OR "cancer urinary bladder"[Title/Abstract] OR "malignant tumor of urinary bladder"[Title/Abstract] OR "cancer of the bladder"[Title/Abstract] OR "bladder cancer"[Title/Abstract] OR "bladder cancers"[Title/Abstract] OR "cancer bladder"[Title/Abstract] OR "cancer of bladder"[Title/Abstract] OR "Urinary Bladder Neoplasms"[MeSH Terms]))

Embase:

Session Results

……………………………………………

No.	Query	Results	Date
#12.	#5 AND #8 AND #11	348	12 Apr 2022
#11.	#9 OR #10	1,295,988	12 Apr 2022
#10.	'placebo':ab,ti OR 'randomized':ab,ti OR 'rct':ab,ti	1,055,669	12 Apr 2022
#9.	'randomized controlled trial'/exp	708,157	12 Apr 2022
#8.	#6 OR #7	50,937	12 Apr 2022
#7.	'mitomycin c':ab,ti OR 'mitomycin-c':ab,ti OR 'mitocin-c':ab,ti OR 'mitocin c':ab,ti OR 'mitocinc':ab,ti OR 'nsc-26980':ab,ti OR 'nsc 26980':ab,ti OR 'nsc26980':ab,ti OR 'ametycine':ab,ti OR 'mutamycin':ab,ti	19,261	12 Apr 2022
#6.	'mitomycin'/exp	48,225	12 Apr 2022
#5.	#3 AND #4	12,940	12 Apr 2022
#4.	'non-muscle- invasive':ab,ti OR 'superficial':ab,ti OR 'nmibc':ab,ti	139,448	12 Apr 2022
#3.	#1 OR #2	109,926	12 Apr 2022
#2.	'neoplasm, urinary bladder':ab,ti OR 'urinary bladder neoplasm':ab,ti OR 'bladder tumors':ab,ti OR 'bladder tumor':ab,ti OR 'tumor, bladder':ab,ti OR 'tumors, bladder':ab,ti OR 'neoplasms, bladder':ab,ti OR 'bladder neoplasms':ab,ti OR 'bladder neoplasm':ab,ti OR 'neoplasm, bladder':ab,ti OR 'urinary bladder cancer':ab,ti OR 'cancer, urinary bladder':ab,ti OR 'malignant tumor of urinary bladder':ab,ti OR 'cancer of the bladder':ab,ti OR 'bladder cancer':ab,ti OR 'bladder cancers':ab,ti OR 'cancer, bladder':ab,ti OR 'cancer of bladder':ab,ti	64,367	12 Apr 2022
#1.	'bladder tumor'/exp	103,073	12 Apr 2022

Cochrane：

Date Run: 12/04/2022 19:15:13

Comment:

ID Search Hits

#1 MeSH descriptor: [Urinary Bladder Neoplasms] explode all trees 1597

#2 (Neoplasm, Urinary Bladder):ti,ab,kw or (Urinary Bladder Neoplasm):ti,ab,kw or (Bladder Tumors):ti,ab,kw or (Bladder Tumor):ti,ab,kw or (Tumor, Bladder):ti,ab,kw or (Tumors, Bladder):ti,ab,kw or (Neoplasms, Bladder):ti,ab,kw or (Bladder Neoplasms):ti,ab,kw or (Bladder Neoplasm):ti,ab,kw or (Neoplasm, Bladder):ti,ab,kw or (Urinary Bladder Cancer):ti,ab,kw or (Cancer, Urinary Bladder):ti,ab,kw or (Malignant Tumor of Urinary Bladder):ti,ab,kw or (Cancer

of the Bladder):ti,ab,kw or (Bladder Cancer):ti,ab,kw or (Bladder Cancers):ti,ab,kw or (Cancer, Bladder):ti,ab,kw or (Cancer of Bladder):ti,ab,kw 5700

#3 #1 or #2 5700

#4 (non-muscle-invasive):ti,ab,kw or (superficial):ti,ab,kw or (nmibc):ti,ab,kw 8418

#5 #4 and #3 1379

#6 MeSH descriptor: [Mitomycins] explode all trees 1319

#7 (Mitomycin C):ti,ab,kw or (Mitomycin-C):ti,ab,kw or (Mitocin-C):ti,ab,kw or (Mitocin C):ti,ab,kw or (MitocinC):ti,ab,kw or (NSC-26980):ti,ab,kw or (NSC 26980):ti,ab,kw or (NSC26980):ti,ab,kw or (Ametycine):ti,ab,kw or (Mutamycin):ti,ab,kw 2238

#8 #7 OR #6 2594

#9 #8 AND #5 296
